# Kenyan Women Bearing the Cost of Climate Change

**DOI:** 10.3390/ijerph182312697

**Published:** 2021-12-02

**Authors:** Elizabeth M. Allen, Leso Munala, Julie R. Henderson

**Affiliations:** Public Health Department, St. Catherine University, St. Paul, MN 55105, USA; jrhenderson660@stkate.edu

**Keywords:** intimate partner violence, climate change, women, Kenya, severe weather, floods, environmental health, IPUMS-DHS

## Abstract

Climate change-induced crises can aggravate intimate partner violence (IPV); the loss of income when weather affects the agricultural industry can exacerbate violence at home. In Kenya, climate change has increased precipitation during the rainy season and raised temperatures during the dry season, resulting in floods and droughts. For 75% of Kenyans, agricultural activities are their primary source of income. This research aims to assess patterns in IPV and severe weather events (SWE). We examined Integrated Public Use Microdata Series-Demographic Health Survey (IPUMS-DHS) data from 2008 and 2014 for IPV severity and frequency. We used Emergency Events Database (EM-DAT) data along with GPS coordinates to identify SWEs (defined as any flood >10 days) by county in Kenya. Overall, women were more likely to experience IPV if their spouse worked in agriculture (Odds Ratio (OR) = 1.22, 95% Confidence Interval (CI): 1.10–1.36). There was a 60% increase in the odds of reporting IPV in counties that experienced an SWE as compared to counties that did not experience an SWE (OR = 1.60, 95% CI: 1.35–1.89). This analysis further supports the growing body of research that suggests a relationship between climate change-related weather events and violence against women.

## 1. Introduction

In the wake of environmental tragedies, efforts focus on immediate healthcare needs and rebuilding infrastructures and economies. The differential effects among men and women, namely, the social and human costs, are often overlooked. Extreme weather patterns can put women in more vulnerable positions and often lead to increases in violence against women. As the number of severe environmental tragedies increases, so will the prevalence of intimate partner violence (IPV).

The pattern of environmentally associated violence has been documented both during and after extreme weather events. For example, rates of gender-based violence increased from 4.6/100,000 per day to 16.3/100,000 per day after Hurricane Katrina in 2005 [1]. Similarly, in the aftermath of Cyclones Vania and Atu that hit Vanuatu, there was a 300% increase in new domestic violence victims seeking care at the Tanna Women’s Counseling Centre [2]. Increasing temperatures significantly reduce well-being [3] and increase overall violence [4]. This is also shown specifically with IPV [5]. In a longitudinal analysis conducted in Spain, the risk of IPV increased by 40% three days after the heatwave. Police reports of violence and helpline calls also modestly increased post heatwave [5].

In many parts of the world, climate change directly affects the lives of women and girls daily, more so than for men and boys. Weather-related chores have been shown to increase women’s risk of sexual assault and domestic violence, decrease their time spent in school, and thus decrease women’s ability to achieve economic independence [6]. For example, in many parts of the world, women bear the responsibility of fetching water for the household. With the increased intensity of droughts, women need to travel further to reach water sources, and the longer travel time increases women and girls’ risk of sexual assault [7]. This increase in travel time can also decrease the time girls have for school, further disempowering women. 

Weather-related disasters because of climate change can place a number of stresses on a family. Crop destruction, loss of livestock, and loss of property resulting in both financial loss and food shortage place a significant burden on those who need to provide for their families [8]. Changing weather patterns have challenged and will continue to challenge farmers’ decisions about when to plant their crops [9]. Moreover, farmers are finding it harder to consistently grow enough food to meet increasing demands [10]. Future predictions for the next century include a wetter rainy season leading to more common and more severe flooding, more severe droughts due to increasing temperatures, and a decrease in both crop yields and livestock production [11].

In this paper, we focus on the relationship between severe weather events in Kenya and the social costs to women. The agricultural industry accounts for an estimated 75 percent of informal employment and is the principal source of rural income and livelihoods [12,13]. The World Bank estimates that most farmers in Kenya, 70–80% of the farming community, rely on small-scale subsistence farms [13]. In 2020, agriculture accounted for 33% of Kenya’s gross domestic product; 20% of Kenya’s landmass is adequate for farming [12]. Irrigation of this cropland is extremely rare, with 98% of this land dependent on rainfall alone [12]. However, since the 1970s, the average rainfall during the long season has decreased by 100 mm.

During periods of economic stress, alcohol consumption often increases, which is directly associated with increases in domestic violence [14,15]. When families are negatively affected by severe weather, men may express anxiety and frustration by directing emotional, physical, and/or sexual violence against their partners [6,8]. Moreover, as small-scale subsistence farmers rely solely on the production of their farms to provide for their families, a natural disaster has detrimental effects on their livelihoods, which can be particularly devastating in Kenya due to such a vast small-scale subsistence farming economy [13]. As we see increases in severe weather events, we will also see increases in intimate partner violence around the globe. 

Severe weather events (SWE) are interrupting the agriculture industry as weather patterns become more unpredictable. The impact of increased SWEs on the agricultural industry leads to increased stress for families along with decreased food affordability, which could result in millions of people reaching an extreme level of poverty [8,16,17]. For populations that routinely struggle with food insecurity, the negative impact is multiplied. The 2014 Kenya Demographic and Health Survey showed high levels of chronic food insufficiency for Kenyans, with 23% of urban household respondents and 36% of rural household respondents acknowledging not having enough food to eat during the seven days prior to the survey [18].

The frequency and intensity of some extreme weather events have increased and will continue to increase as a consequence of global climate change [19]. Some of these changes include a decrease in cold temperature extremes, an increase in warm temperature extremes, an increase in extreme high sea levels, and an increase in the number of heavy precipitation events in a number of regions. These changes impact ecosystems and food security. Climate change will amplify existing risks and create new risks for natural and human systems [19]. This analysis aims to explore the relationship between severe weather events and intimate partner violence among Kenyan women. 

## 2. Materials and Methods

Since the 1980s, the Demographic and Health Surveys (DHS) have been the main source of information about health and well-being for women and young children in low- and middle-income countries (LMICs). Since most LMICs conduct DHS surveys at 3- to 5-year intervals, DHS data can be used to study trends over time, and new topics (e.g., HIV testing and awareness) are added in response to emerging public health crises (e.g., the HIV pandemic). Since 2000, many Demographic and Health Surveys have included questions about intimate partner violence—emotional, physical, and sexual. In addition, many Demographic and Health Surveys release GPS coordinates for the approximate location of sampling clusters, allowing researchers to link outside data sources indexed by geographic location—to, for example, information on extreme weather events—with DHS survey participants [20].

The DHS surveys are intended to represent the total population of a nation. Sampling uses a multi-stage process to select households to be surveyed. The country is initially stratified by geographical regions, which are then further stratified into urban/rural. Clusters within each region are selected, and within these clusters, 25–30 households are randomly selected to interview [21].

For our analysis, we used harmonized DHS data from Integrated Public Use Microdata Series (IPUMS), which recodes variable names and codes to be consistent across time and space and supplies supplementary documentation about variables [22]. We used data from Kenya for survey years 2008 and 2014, along with the GPS location data collected for each survey cluster. GPS data are randomly displaced to provide for the confidentiality of the respondents. Urban clusters are displaced from 0–2 kms, and rural clusters are displaced from 0–5 kms, with an additional 1% of the rural clusters having a displacement of up to 10 kms. The displacement ensures the GPS coordinates of the cluster remain within the boundaries of the survey region [20].

The 2008 and 2014 Kenya DHS surveys randomly selected one woman from each household, who was of childbearing age (age 15–49) and had ever been married or lived with a man, to complete the Domestic Violence (DV) questionnaire. The DHS surveys use the broad term “domestic violence” to refer to violence experienced from a partner or husband and violence from other family members; we will be referring to violence from a partner or husband specifically as IPV. The questionnaires asked respondents whether they had experienced IPV and how often it occurred in the past 12 months (often, sometimes, or not at all). The survey questions addressed 12 different forms of IPV in the areas of emotional, sexual, and physical violence. All women who responded to at least one of the 12 IPV questions were included in this analysis. These variables were recorded into a single dichotomous variable. Those who responded with never or not at all in the past year on all 12 questions were coded as “0”, and those who responded with occurred often or sometimes in the past year on at least one of the 12 IPV questions were coded as “1”. If a woman’s response to any single question was unclear, the response was reported as “missing” for that variable. Appendix A includes the survey questions and response choices. 

For the 2008 survey, Kenya was divided into 8 provinces (Central, Coast, Eastern, Nairobi, North Eastern, Nyanza, Rift Valley, and Western). Following the 2010 constitution, Kenya was reorganized into 47 counties, resulting in a different division of regions for the 2014 surveys. We converted the 8 provinces from the 2008 survey to match the 47 counties in 2014 using latitude and longitude coordinates in [23].

We used data from the Centre for Research on the Epidemiology of Disasters (CRED) Emergency Events Database (EM-DAT) to identify severe weather events in Kenya [24]. We identified weather events in the 2 years prior (2006, 2007, 2012, 2013) and the year of (2008, 2014) DHS data collection. For analysis purposes, we defined a severe weather event as a flood lasting 10 or more days. Though droughts are also reported by EM-DAT and can have a detrimental effect on agriculture, only two droughts were reported in the study period, and thus, droughts were not evaluated in this analysis.

All statistical analyses were conducted using StataC 16 [25]. We conducted a logistic regression with a mixed-methods model, grouped by the 47 counties in Kenya to estimate the odds ratios (OR) and 95% confidence intervals (CI) for the association between living in a county that experienced an SWE and reporting experiences of IPV. Experiences of IPV were examined in an overall analysis (any reports of IPV in the past 12 months) and IPV specific analyses (emotional, physical, and sexual IPV). We then evaluated the relationship between a change in severe weather and experiences of IPV. We looked at the year prior to DHS data collection and the year of DHS data collection and created a variable indicating if SWEs went up, went down, or stayed the same in each county. Each of the final models was adjusted for urban/rural residence, if the woman’s partner works in the agricultural industry, and if the partner drinks alcohol.

## 3. Results

The 2008 survey included 4903 women, and the 2014 survey included 4512 women. The mean age of the study population was 28.4 years. The majority of women (approximately 80% in both years) were married. More than half (54%) achieved a primary level of education, while just under 20% of the study population had received no formal education. Complete demographic information on the study population can be found in Table 1. 

**Figure 1:**
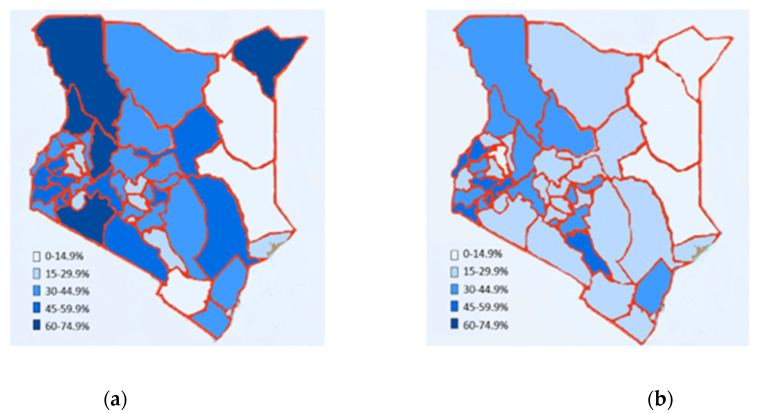
Prevalence of Interpersonal Violence per County in the Demographic and Health Surveys (DHS) Survey Years: (**a**) 2008; (**b**) 2014.

In 2014, Kenya experienced a decrease in the number of women reporting IPV within the last year compared to 2008. In 2008, the prevalence of IPV ranged from a low of 8.6% in Wajir to a high of 68.1% in Baringo. The prevalence in 2014 was altogether lower, with a low of 6.3% in Garissa and a high of 54.1% in Bungoma. Figure 1 is a comparison by county of the prevalence of IPV for 2008 and 2014. 

There were greater odds of reporting IPV among women whose partners worked in agriculture as compared to women whose partners did not work in agriculture (OR = 1.22, 95% CI: 1.10–1.36). There were also greater odds of reporting IPV in counties that experienced a severe flood when compared to counties that did not experience a severe flood (OR = 1.60, 95% CI: 1.35–1.89). Additionally, there was a strong relationship between a partner drinking alcohol and reporting all forms of IPV (OR =2.38, 95% CI: 2.17–2.62). These associations held true in both crude and adjusted models. Though living in a rural location was not associated with reporting IPV, rural residence was highly correlated with a partner working in the agricultural industry.

A change in the number of floods experienced by a county was associated with a change in the prevalence of IPV. If there was an increase in the number of floods during the survey year from the year prior to the survey year, there was an increase in the odds of reporting IPV (OR = 1.74, 95% CI: 1.43–2.13). This held true for both physical violence (OR = 2.01, 95% CI: 1.63–2.49) and sexual violence (OR = 1.48, 95% CI: 1.07–2.05) but not emotional abuse (OR = 1.03, 95% CI: 0.82–1.30). Table 2 provides complete results.

## 4. Discussion

Our findings suggest that an increase in SWEs is correlated with an increase in physical and sexual violence. This same increase was not seen with emotional violence. Further studies examining this observation would be beneficial when focusing on interventions to decrease IPV. We identified a relationship between experiences of IPV and SWEs in Kenya during the study time period. There was a 1.6 increase in IPV odds among those who lived in counties that experienced an SWE. The results of this work further support the growing body of research that suggests a relationship between climate change-related weather events and violence against women [6,8,16,26]. The current body of literature has found that women are adversely affected by climate change, which has been documented during and after a number of climate events, including heatwaves, hurricanes, and cyclones [1,2,5]. As noted earlier by Gevers et al. (2020), the physical demands on women increase with climate change. In addition, as was noted by UN Women, an increase in cyclone activity was correlated with an increase in seeking DV counseling. Our findings support previous literature and further the knowledge of the impacts of SWEs on women.

Previous studies have shown a correlation between domestic violence and the consumption of alcohol by a male partner. A study among pregnant women in Kenya noted that those who experienced partner violence were more likely to have a partner who consumed alcohol [14,27]. Similarly, Camey et al. noted an increase in alcohol use among men in Australia coping with stress due to droughts (2020). The compounding effect of climate change and alcohol consumption places women who have a husband working in agriculture at higher risk for experiencing IPV. Our results support this body of research because we identified a 2.38 increase in odds of IPV among those whose partners consumed alcohol.

IPV has progressively become a prominent issue both within Kenya and globally. Gender-based violence (GBV) is any act against a person based on their gender. According to UN Women (n.d.), GBV is a violation of basic human rights, and IPV has now become a human rights issue that has changed from a private concern within a family to a public concern. The Constitution of Kenya 2010 states that all people should be free from any form of violence, and non-action against violence is equal to accepting it, which perpetuates its existence [28]. In 2015, the United Nations General Assembly set the Sustainable Development Goals (SDG), SDG 5.2: eliminate all forms of violence against all women and girls in public and private spheres, including trafficking and sexual and other types of exploitation.

There were major differences in the prevalence of reported IPV across regions, differences that merit further study to determine whether their cause is economic, a variation across ethnic groups, or due to some other factor. In 2014, Kenya experienced a decrease in the number of women reporting IPV within the last year compared to 2008. This decrease may be due, in part, to the legislation change and the changing attitudes of men and women regarding IPV. In the Kenyan DHS surveys, women and men were asked questions regarding their attitudes towards IPV (i.e., views on when a husband is justified in hitting or beating his wife). Between 2008 and 2014, there was a decrease in the percentage of both women (29.8 to 24.9) and men (22.4 to 19.0) who consider hitting or beating a woman to be justified in some circumstances. The odds of a woman justifying beating or hitting were greater among women who experienced IPV in the past year compared to those who did not experience IPV. This change in attitude and the decrease in the number of women reporting IPV may be related to the Kenyan legislation change (as embodied in the new Constitution) that made IPV a public concern.

A number of limitations should be considered when interpreting the results of this study. The extreme weather events may have been more localized in their effects than the geographic units we used in our analysis. For example, flooding may only have occurred on agricultural land that was near a body of water (river or coastline), while the rest of the county was not affected. It is possible that women in the areas affected by flooding in a county were more likely to experience IPV, but the effect is diluted if most of the land in the county was not affected by flooding. More advanced research methods allowing for more precise location of floods and respondents would provide more accurate information. The measures of IPV were based on self-reporting and, thus, potentially underreported, which may have underestimated our results. The variables analyzed for this research were unique to the 2008 and 2014 Kenyan data sets, which limited the number of years available for analysis. While the county variation in IPV rates was not cross-validated with police reports, studies [29,30] suggest that rural women in Kenya and elsewhere do not report IPV cases. Similarly, this could have led to an underestimation of our final results. The next DHS survey for Kenya should be completed in 2021. This analysis could be rerun using data from this survey along with severe weather events to determine if the findings hold true. However, despite these limitations, this analysis has a number of strengths, including representative sampling across Kenya, a large sample size that allowed for adequate control of potential confounding variables, and a comprehensive questionnaire that included 12 measures of IPV.

## 5. Conclusions

Women are key stakeholders when it comes to climate change and violence. Over half of Kenyan rural women experience some form of IPV in their lifetime [31]. Moreover, climate change unequally impacts women due to their lower socioeconomic status and not being accorded the same basic human rights as their male counterparts [32]. Women need to be included at the decision-making table when developing interventions to combat climate change and increase protections for women. In Kenya, parliament is composed of 78% men and 22% women [33]. This imbalance of decision-making capacity needs to be rectified for women to take an equal voice in climate change.

Climate change and the resulting severe weather events continue to challenge the world [34]. As these challenges persist, interventions to both increase protection for women and raise women’s status are needed to mitigate women’s risk. Climate action is an essential component in the ongoing fight to eliminate violence against women and girls. This analysis adds to the urgency of addressing gender-based violence in all forms alongside action to stop environmental degradation and gender-based violence and demonstrates that the two issues often need to be addressed together.

## Figures and Tables

**Table 1:** Demographic Characteristics of Study Population.

Characteristics	Survey Year 2008 (*n* = 4903)	Survey Year 2014 (*n* = 4512)
	N (%)	N (%)
Urban/Rural Status		
Urban	1396 (28.5)	1642 (36.4)
Rural	3507 (71.5)	2870 (63.6)
Age		
15–19	220 (5.5)	153 (3.4)
20–29	2062 (42.1)	1898 (42.1)
30–39	1658 (33.8)	1580 (35.0)
40–49	963 (19.6)	881 (19.5)
Marital Status		
Married	3952 (80.6)	3583 (79.4)
Living Together	316 (6.5)	278 (6.2)
Divorced/Widowed	367 (7.4)	357 (7.9)
Other	268 (5.5)	296 (6.5)
Highest Educational Level		
None	937 (19.1)	788 (17.5)
Primary	2647 (54.0)	2451 (54.3)
Secondary or higher	1319 (26.9)	1273 (28.2)
Currently Working		
Yes	2950 (60.2)	2984 (66.1)
No	1942 (39.7)	1525 (33.8)
Missing	11 (0.2)	3 (0.1)

**Table 2:** Odds of Having Experienced Intimate Partner Violence by Severe Flood Events N = 9415 for 47 County Data.

	All Forms IPV		Emotional IPV		Physical IPV		Sexual IPV	
	**OR**	**(95%CI)**	**OR**	**(95%CI)**	**OR**	**(95%CI)**	**OR**	**(95%CI)**
Unadjusted:								
Severe Flood	1.56	(1.31,1.85)	0.90	(0.74, 1.09)	1.86	(1.55, 2.23)	1.57	(1.19, 2.07)
Partner works in agriculture	1.25	(1.13, 1.38)	1.22	(1.09, 1.36)	1.31	(1.18, 1.46)	1.26	(1.07, 1.46)
Partner drinks alcohol	2.37	(2.16, 2.61)	2.31	(2.08, 2.55)	2.56	(2.31, 2.84)	2.70	(2.33, 3.12)
Urban/Rural residence	1.05	(0.95, 1.16)	0.94	(0.84, 1.05)	1.17	(1.04, 1.31)	1.02	(0.87, 1.20)
Adjusted models:								
Severe Flood	1.60	(1.35, 1.89)	0.92	(0.76, 1.12)	1.91	(1.59, 2.29)	1.61	(1.22, 2.12)
Partner work in agriculture	1.22	(1.10, 1.36)	1.24	(1.10, 1.39)	1.25	(1.12, 1.40)	1.22	(1.04, 1.44)
Partner drinks alcohol	2.38	(2.17, 2.62)	2.30	(2.08, 2.55)	2.58	(2.33, 2.86)	2.71	(2.34, 3.13)
Urban/Rural residence	0.95	(0.85, 1.06)	0.87	(0.77, 0.98)	1.05	(0.93, 1.18)	0.92	(0.77, 1.09)
Change in Flood Pattern								
Increase (0 to 1)	1.74	(1.43, 2.13)	1.03	(0.82, 1.30)	2.01	(1.63, 2.49)	1.48	(1.07, 2.05)
Decrease (1 to 0)	0.78	(0.66, 0.93)	0.89	(0.74, 1.07)	0.67	(0.56, 0.81)	0.74	(0.58, 0.95)
Stay Same (1 to 1)	0.80	(0.54, 1.19)	0.56	(0.37, 0.85)	0.86	(0.57, 1.31)	1.27	(0.71, 2.28)

## Data Availability

Publicly available datasets were analyzed in this study. The data can be found at IPUMS-DHS: https://www.idhsdata.org/idhs/index.shtml and EM-DAT: https://www.emdat.be/ (accessed on 10 November 2021).

## Appendix A. DV Questions from 2008–2009 and 2014 DHS Survey

### Appendix A.1. DV Questions Pre-Survey Statement

Now, I would like to ask you questions about some other important aspects of a woman’s life. You may find some of these questions very personal. However, your answers are crucial for helping to understand the condition of women in Kenya. Let me assure you that your answers are completely confidential and will not be told to anyone and no one else in your household will know that you were asked these questions.

Currently Married/Married Living with A Man ____ (respondents taken to DV from partner questions).

Formerly Married/Lived With A Man (Read In Past Tense And Use ‘Last’ With Husband/Partner) (respondents taken to DV from partner questions).

Never Married/Married Never Lived with A Man (respondents taken to DV from other people).

### Appendix A.2. Now I Need to Ask Some More Questions about Your Relationship with Your (Last) (Husband/Partner). Did Your (Last) (Husband/Partner) Ever

(a) Say or do something to humiliate you in front of others?
(b) Threaten to hurt or harm you or someone you care about?
(c) Insult you or make you feel bad about yourself?
(d) Push you, shake you, or throw something at you?
(e) Slap you?
(f) Twist your arm or pull your hair?
(g) Punch you with his fist or with something that could hurt you?
(h) Kick you, drag you, or beat you up?
(i) Try to choke you or burn you on purpose?
(j) Threaten or attack you with a knife, gun, or any other weapon?
(k) Physically force you to have sexual intercourse with him even when you did not want to?
(l) Force you to perform any sexual acts you did not want to?

### Appendix A.3. For Each of the above Questions a–l That a Woman Answered Yes, They Were Then Asked This Follow-Up Question

How often did this happen during the last 12 months: often, only sometimes, or not at all?

(1) Often
(2) Sometimes
(3) Not at all
